# Stem Cell Therapy as a Treatment for Osteogenesis Imperfecta

**DOI:** 10.1007/s11914-020-00594-3

**Published:** 2020-07-24

**Authors:** Cecilia Götherström, Lilian Walther-Jallow

**Affiliations:** Department of Clinical Science, Intervention and Technology, Division of Obstetrics and Gynecology, Karolinska Institutet, ANA Futura, floor 8, Alfred Nobels Allé 8, 141 52 Huddinge, Stockholm, Sweden

**Keywords:** Osteogenesis imperfecta, Brittle bones, Stem cell therapy, Stem cell transplantation, Prenatal therapy, Mesenchymal stem cells

## Abstract

**Purpose of Review:**

Osteogenesis imperfecta (OI) is a chronic disease with few treatment options available. The purpose of this review is to provide an overview on treating OI with mesenchymal stem cells (MSC).

**Recent Findings:**

Off-the-shelf MSC have a good safety profile and exhibit multilineage differentiation potential and a low immunogenic profile and are easy to manufacture. Their ability to migrate, engraft, and differentiate into bone cells, and also to act via paracrine effects on the recipient’s tissues, makes MSC candidates as a clinical therapy for OI. Due to their high osteogenic potency, fetal MSC offer an even higher therapeutic potential in OI compared with MSC derived from adult sources. Preclinical and initial clinical data support the use of MSC in treating OI.

**Summary:**

The characteristics of MSC make them of great interest in treating OI. MSC may be safely transplanted via intravenous administration and show potential positive clinical effects.

## Osteogenesis Imperfecta

One child among 10,000 to 20,000 is born with osteogenesis imperfecta (OI), a group of genetic disorders caused by > 1400 different dominant and > 150 recessive known mutations, and new mutations continue to be found [1, 2]. Mutations in the collagen genes resulting in abnormal collagen microfibril assembly are the most common. The major clinical manifestations are atypical skeletal development, osteopenia, multiple painful fractures, and short stature, but individuals with OI may also suffer from other disorders including brittle teeth, hearing loss, and hypermobile joints. Throughout their lifetime, they also have a higher risk of pulmonary problems, heart disease (including valve insufficiency and aneurysms) that usually becomes evident only in later childhood or adulthood, and bleeding and coagulation deficiencies. Life expectancy is not affected in milder OI types but may be shortened for those with more severe types [3]. OI presents in a clinically heterogeneous manner, ranging from the mild Type I to lethal Type IIA/C [1, 2]. In 1979 OI was classified by Sillence et al. into four groups (Types I–IV) according to clinical, radiological, and histological findings [4, 5]. Type I OI is the mildest form and may involve only a few fractures over a person’s lifetime, whereas Type IIA/C OI is lethal. Type III OI is the most severe form that is compatible with survival to adulthood. Individuals affected by Type III OI may experience hundreds of fractures in a lifetime. Most commonly, OI presents in childhood with multiple fractures after little or no trauma (Type I/mild Type IV). Type IV can be moderately severe but are very variable. The growing knowledge about the genetic cause of OI have challenged the first classification by Sillence et al., and new classifications have been proposed in 2004, 2007, and 2010 [6–8]. More than 17 proteins have been described that interact directly or indirectly with the biosynthesis of collagen, and a mutation in one of these genes results in rare forms of mostly autosomal recessive OI, which share the phenotypic features of the classical OI Types I–IV. Therefore, the first classification by Sillence and colleagues has been expanded with OI Types V–VIII and includes other forms of OI based on the underlying mutations [8]. The first classification system is currently used as a grading of clinical severity of OI.

No cure for OI exists, and current treatments do not address the underlying molecular pathology. The goal of treatment is to increase overall bone strength to prevent fractures, maintain mobility, promote normal function, and improve the quality of life. This goal is accomplished with physiotherapy to strengthen the muscles and improve mobility and lifelong orthopedic interventions such as rods in the long bones for correcting bone deformities. Bisphosphonates are used to reduce bone resorption and increase bone mineral density. The effect of bisphosphonates in OI has been debated, and according to a recent systematic review, randomized controlled trials did not demonstrate a significant improvement in function and mobility with oral bisphosphonate administration, whereas nonrandomized open-label uncontrolled studies demonstrate that oral and intravenous bisphosphonate administration objectively improved function and mobility [9].

## Stem Cells as a Treatment of Osteogenesis Imperfecta

Given the severe clinical manifestations of OI and the limitations of current treatments, a clear unmet medical need exists for treatment of OI. Transplantation of mesenchymal stem cells (MSC) presents a potential new mode of treatment, specifically starting treatment before birth or as early as possible after birth, to prevent irreversible damage (see information about the BOOSTB4 trial below).

MSC are stromal cells, originally identified in adult bone marrow, and display colony-forming capacity and are non-hematopoietic and non-endothelial. They are multipotent cells that can be easily expanded and can differentiate into a variety of cell types including osteoblasts, chondrocytes, myocytes, and adipocytes [10]. In immunocompetent allogeneic/xenogeneic animal models, intravenously infused MSC engraft widely in multiple tissues and demonstrate site-specific differentiation [11–14]. In addition, MSC constitutively secrete an extensive array of factors and microvesicles that may act in a paracrine fashion in vivo [15, 16].

## Advantages of Fetal-Derived MSC for Treatment of OI

Fetal MSC are found at a higher frequency per nucleated cell and have a greater colony-forming capacity than adult MSC. First-trimester fetal MSC demonstrate higher proliferative capacity and have longer telomeres, a superior proliferative potential, and cycle faster than adult MSC [17]. However, fetal MSC do senesce and stop dividing within the Hayflick limit (defined as the number of times a normal human somatic cell will divide before cell division stops) but can be expanded for more population doublings than adult MSC before senescing [18, 19]. Apart from the mesodermal lineages, fetal MSC possess the ability to differentiate to muscle cells, and oligodendrocytes [13, 20–23], and differentiate more readily into bone than adult MSC [19, 24, 25]. Upon osteogenic differentiation, fetal MSC produced more robust osteogenic genes and induced more calcium production in vitro and reached higher levels of osteogenic gene upregulation both in vivo and in vitro than adult MSC [24]. In another direct comparison, fetal first-trimester MSC were compared with MSC derived from the term umbilical cord, adult adipose tissue, and adult bone marrow. It was shown that all MSC had equivalent immune-phenotype but that the fetal MSC exhibited the greatest osteogenic capacity [19].

In principle, allogeneic cells are subject to HLA-mediated elimination from the circulation by the host’s immune system. MSC, however, have low immunogenicity, and fetal MSC are potentially even less immunogenic than adult MSC. Both fetal and adult MSC express intermediate levels of HLA class I on the surface, but adult MSC express these antigens at slightly higher levels than fetal MSC do [17, 19, 26–28]. MSC possess immunomodulatory properties demonstrated by their ability to inhibit lymphocyte proliferation, but fetal MSC are not as immunomodulatory as adult MSC and mainly inhibit mitogen-activated lymphocytes [26, 27, 29, 30]. If MSC are stimulated with IFNγ for full expression of human leukocyte antigen (HLA) class II, the major transplantation antigen, they still escape recognition by alloreactive T cells [26, 27], and MSC differentiated to osteogenic and adipogenic lineages are also non-immunogenic [26, 27].

## Preclinical Models on MSC Treatment of OI

Nonclinical pharmacological studies of MSC demonstrate their potential for treatment of OI. The utility of human fetal MSC for bone regeneration was demonstrated in a rat model of critical-size bone defects treated with implant of tissue-engineered bone graft seeded with fetal human MSC [25]. The MSC tissue-engineering implant group had better bone formation, more compact and woven bone, stiffer bone, and a larger vasculature network in the defect area than the control group and the group implanted with only an acellular scaffold. Studies in mouse models of OI have shown successful engraftment of human adult MSC, human fetal MSC, and mouse MSC transplanted pre- or postnatally [31–35]. The human fetal MSC were particularly detected in the bones and at sites of bone formation and remodeling and fracture healing sites, and the transplanted cells differentiated into mature osteoblasts expressing osteocalcin and producing collagen COL1a2 protein [35]. Upregulation of endogenous genes involved in early stages of endochondral and intramembranous ossification, and late hypertrophic chondrocyte differentiation was also observed [34]. Transplantation of human fetal MSC resulted in reduced bone brittleness with a decrease in long bone fracture rates by 67% to 84%. It also lead to increased bone thickness, volume, stiffness, and strength and increased collagen and mineral content and decreased hydroxyproline content in bones [34, 35]. Normalization of growth plate height was also observed [35].

## Safety and General Clinical Knowledge of MSC

In completed and ongoing clinical trials, intravenous infusion of autologous or allogeneic MSC appears safe and well tolerated. Because of these characteristics, MSC (mostly adult) have been tested in clinical trials for a diverse variety of disorders [36, 37••, 38••]. In these trials, thousands of patients have received treatment, mostly with MSC derived from adult sources, and few adverse events have been reported. A 2012 meta-analysis of the randomized clinical trials did not detect an association with acute infusion toxicity, organ system complications, infection, death, or malignancy although a significant association between MSC and transient fever was observed after up to 90 months of follow-up [36]. The meta-analysis included both autologous and allogeneic MSC as well as expanded/cultured cells. A concern within the field has been the risk for tumor formation in the treated patients. Upon preparation for the clinical BOOSTB4 study described below, several batches of clinical grade fetal MSC were tested for and showed no tumor development in a mouse model, displayed a normal karyotype after expansion, did not express pluripotent-associated markers, and did not grow anchorage-independently. Further, the cells stopped dividing at passages 15–17 (36–48 population doublings) which is in line with the Hayflick limit for somatic cells. Hence, the risk for tumor formation can be considered to be very low. Further, no immunogenic, immunomodulatory, or tumorigenic effects relevant for clinical studies were found in preclinical animal studies.

## Advantages of Early Treatment of Osteogenesis Imperfecta

### Early Childhood Treatment

Initiation of treatment with MSC of severe types of OI is desirable, as early as possible before additional pathology occurs, in order to achieve the objectives outlined in Table 1. With early treatment the rapid growth of the baby/child provides an opportunity for engraftment, expansion, and subsequent migration and distribution of the donor cells to different anatomical compartments. The effect of MSC may be enhanced with early treatment because a lesser amount of poor quality OI type collagen that requires replacement has been produced. Small numbers of donor cells result in a large amount of healthy collagen: 2% engrafted donor cells led to 20% healthy collagen in the bones in a mouse model of OI [32].

**Table 1:** Objectives for early initiation of MSC treatment of severe OI

**Reasons for initiation of early treatment with MSC**	
**Desired effect**	**Description**
Reduction of detrimental effects on respiration	Improve/arrest the abnormal chest wall and spine architecture (kyphoscoliosis, scoliosis, rib fractures, pectus deformities, and abnormalities of lung collagen)
Improvement in healing of fractures	Enhanced fracture healing will reduce negative effects of fractures, alleviate pain, and improve the quality of life for individuals with OI
Reduction of risk of additional fractures	Reduced frequency of fractures will diminish negative effects of fractures, alleviate pain, and improve the quality of life for individuals with OI
Reduction of risk of bowing of long bones	Reduced risk of bowing decreases the need for orthopedic surgical procedures and hospital stay, improves the mobility, alleviates pain, and improves the quality of life for individuals with OI
Improvement in growth	Increased/not plateaued growth will improve the mobility and the quality of life for individuals with OI
**Additional reasons for initiation of prenatal treatment with MSC**	
**Desired effect**	**Description**
Better physiological conditions for systemic distribution of the administered cells	As the administration is given into the umbilical vein in the fetus, it will bypass the pulmonary circulation via two fetal shunts, the ductus arteriosus, and the foramen ovale. This ensures that the administered MSC go directly into the systemic circulation and can then home directly to the bones. MSC administration after birth is performed into a peripheral vein, with many MSC becoming trapped in the microcirculation of the lungs before fewer MSC reach the systemic circulation
Better distribution and engraftment of the cells	Fetal life is a time of natural stem cell proliferation and migration to different anatomic compartments. The rapid growth of the fetus provides an opportunity for higher engraftment, wider expansion, and subsequent migration and distribution of the donor cells
Better effect of engrafted cells	The effect of MSC may be enhanced with prenatal treatment because a lesser amount of poor quality OI type collagen that requires replacement has been produced. As described above, small numbers of donor cells result in a large amount of healthy collagen
Naïve immune system	The relatively naive fetal immune system may permit the development of immune tolerance towards donor cells
Psychosocial value	A better psychosocial situation for the mother and father may result from the birth of a child who has already been treated
Improved parent and family quality of life	Having a child with chronic disease significantly affects the parents and the families quality of life, which includes both psychological and physical quality of life
**Additional theoretical benefits of MSC treatment**	
**Improvement of**	**Reduction of**
Bone mineral density	Platyspondyly (vertebral fractures)
Motor milestones	Pain
Quality of life	Hospital stay

### Prenatal Treatment

Fetuses affected by OI suffer from the disease even during fetal life. The natural history of the disease during fetal life is that fractures continue to develop and can be observed on ultrasound imaging as gestation advances. Thus, there is a rationale for treatment to begin as early as possible and thereby ameliorate pathology that has begun already during fetal life, or even prevent fractures from occurring. Apart from the objectives described in the first part of Table 1, additional benefits of initiation of treatment of severe types of OI during fetal life compared with postnatally are described in the second part of Table 1.

## Clinical Experience of Stem Cell Treatment of Osteogenesis Imperfecta

Postnatal stem cell transplantation for the treatment of OI has been attempted in children diagnosed with severe OI. The first clinical proof of principle came from Horwitz et al. in which six children with OI Type III were transplanted with HLA-matched whole bone marrow donated by a sibling [39]. The linear growth increased from 1.25 cm at 6 months before compared with 7.5 cm at 6 months after transplantation. During the same time period, the fracture frequency was reduced by 80% in spite of low-level (< 2%) donor osteoblast engraftment. In a follow-up trial [40], MSC were isolated from the bone marrow donors, culture expanded, and administered intravenously to the recipients. Two doses of MSC showed similar results as the bone marrow transplantation with low (1–2%) donor cell engraftment in the bones and an acceleration of the growth velocity of 60–94% [40]. As explained above, data from mouse studies suggest that significant amounts of normal collagen can be deposited by a relatively small population of donor cells [32], which can explain the marked improvements despite low-level engraftment. No severe adverse events were noted, but one adverse event was noted in one patient, an easily treated urticarial rash after the second MSC infusion. No donor cell engraftment or clinical effect was observed in this patient.

Two case studies of prenatal transplantation of fetal MSC for the treatment of OI Type III and IV have been published [41, 42••]. Both children subsequently received postnatal booster infusions of MSC from the same donor because of a deteriorating clinical picture. While it is difficult to definitively determine the effect of MSC transplantation on OI, the results from these two heterogeneous cases suggest that prenatal MSC transplantation with postnatal booster transplantation is safe and provides a potential clinical benefit, particularly in comparison with untreated individuals with the same mutation. The two cases are described in more detail below.

The first case, a Swedish patient diagnosed with OI, received one prenatal transplantation in 2002 [41]. Ultrasound examinations in gestational weeks 15 to 27 showed that all the limbs were below the fifth percentile with angulated and fractured femur bones. The karyotype was normal. A clinical diagnosis of severe Type III OI was made. She received a dose of 5.0 × 10^6^ HLA-unmatched human first-trimester liver-derived MSC/kg estimated fetal body weight into the intrahepatic part of the umbilical vein at gestational week 32. The procedure was well tolerated by the fetus and the pregnant woman. No fractures occurred after the MSC transplantation, and the fetus followed her growth curve, and the rest of the pregnancy was uneventful. The girl was delivered by caesarean section at gestational week 35 after spontaneous rupture of the membranes. The clinical examination after birth showed typical signs of severe OI with generalized osteopenia, platyspondyly, Wormian skull bones, thin gracile bowed long bones with deformities indicating healed fractures, and an actual fracture of the right femoral diaphysis. Bisphosphonate (pamidronate) treatment was initiated at 4 months of age due to generalized osteopenia and vertebral compression fractures.

Generally, the girl’s clinical course has been better than expected from her genetic mutation. Three other individuals are known to have an identical COL1A2 mutation as this child. One child from Canada is described in Götherström et al. [42••] and presented with a very severe phenotype of OI. This patient did not receive MSC infusion and succumbed at 5 months of age due to respiratory problems despite postnatal bisphosphonates therapy. The two other individuals with the same mutation reported in the literature are described as severe OI (Type II/III). Treatment received by these individuals and other factors affecting their disease are not known. Until 8 years of age, the girl who received MSC was doing reasonably well with approximately one fracture and one compression fracture clinically confirmed every year (5 femoral, 2 clavicular, 1 shoulder and 1 skull fracture, and 11 vertebral compression fractures). She walked with support at 15 months of age and without support at 2 years and 4 months. Remarkably she continued growing at a normal rate, albeit around -5 SD from birth to 6 years of age, deteriorating to -6.5 SD at 8 years of age. At this point the patient received a booster dose intravenously with 2.8 × 10^6^/kg same-donor MSC [42••]. This decision was made based on an increased rate of fractures and the plateaued growth. The following 2 years, the patient did not experience any new fractures, and the mobility and linear growth (-6 SD) improved. Between ages 11–13, the patient received one same-donor MSC booster dose/year to improve her height. She is now 17 years old and is doing well.

The second case was a fetus with OI Type IV who presented with short long bones (< fifth centile) and multiple new and healing fractures at 26 weeks of gestation [42••]. The fetus was transplanted with 30 × 10^6^/kg HLA-unmatched human first-trimester liver-derived MSC at 31 weeks of gestation. The fetus and pregnant woman tolerated the procedure well, and the fetus did not suffer any new fractures for the remainder of the pregnancy or during infancy. Genotyping of the patient and family members identified an autosomal dominant mode of inheritance. Bisphosphonate therapy was initiated from 1 month of age due to poor mineralization. The patient followed her own growth curve just below the third percentile until 1 year of age, when the longitudinal length plateaued. A postnatal intravenous booster dose with same-donor MSC was performed at 1 year and 6 months of age, which was followed by a resumption of her lengthwise growth. She started to walk shortly after the booster dose. The girl is today 10 years old.

Donor cell engraftment has been confirmed in the first case and is limited to the bones [41, 42••]. The detected level of engraftment varies but is consistently low; between 0.003 and 16.6%. Samples from the bone have not been possible to retrieve from the second case.

The two cases summarized above received intravenous doses of fetal MSC prenatally with same-donor MSC booster dose(s) postnatally. There were no signs of any early or late adverse events (follow-up more than 10 and 17 years). The donor cells did not induce any alloreactivity among the patient’s lymphocytes in vitro. Before the booster doses, extensive analysis showed the absence of antibodies directed toward human leukocyte antigen class I and II, IgG and IgM, or fetal bovine serum or a cell-mediated response toward the donor MSC.

## A Clinical Trial Investigating MSC as a Treatment of OI

Boost Brittle Bones Before Birth (BOOSTB4) is an European trial that investigates MSC transplantation as a therapy for severe vital forms of OI (Type III and severe Type IV). The primary endpoint of the phase I/II multicenter trial is safety and tolerability for the infant, the pregnant woman, and the fetus. Secondary endpoints relate to efficacy (fracture frequency, time to fracture, number of fractures at birth, growth, bone mineral density, biochemical bone turnover, and clinical OI status). The overall experience, impact, and perception of the therapy also will be evaluated.

The study will include three groups:

1. Four postnatal doses of 3 × 10^6^ MSC/kg body weight (*n*=15)
2. One prenatal and three postnatal doses of 3 × 10^6^ MSC/kg body weight (*n*=15)
3. Historical and prospective controls (*n*=30–150)

All participants who are eligible can receive MSC, i.e., there is no randomization into the groups. We have established a European network centered in Stockholm in Sweden, London in the UK, Cologne in Germany, and Utrecht/Leiden in the Netherlands. Ethical and regulatory approval has so far been obtained in Sweden and the UK, and the trial is open in Sweden. The BOOSTB4 consortium welcomes participants for inclusion in the clinical trial. Contact the BOOSTB4 consortium for more information: https://ki.se/clintec and https://www.boostb4.eu.

## Future Outlook

It is desirable that research groups within the cell therapy area join forces and develop common programs including guidelines regarding indications, inclusion, and manufacture of cell products. Furthermore, methods for cell transplantation, monitoring, and structured follow-up of involved fetuses and children should be collective in order for future studies to be set-up with the most optimal design. If future cell therapies involving children and pregnant women and their fetuses are not performed according to the highest of standards, there is an obvious risk that the whole field will be fragmented and thus will prevent development within this area.

## Conclusions

At the moment MSC transplantation before and after birth to treat OI is an experimental therapy about to be systematically tested in the international multicenter phase I/II clinical trial BOOSTB4 that will assess the safety and efficacy of fetal MSC transplantation for the treatment of severe types of OI.
